# Rapid identification of antibiotic residues in bovine kidney using coated blade spray-mass spectrometry

**DOI:** 10.1007/s00216-024-05605-1

**Published:** 2024-10-22

**Authors:** Josha Jager, Sjors Rasker, Ane Arrizabalaga-Larrañaga, Rita Boerrigter-Eenling, Michel Rapallini, Marco Blokland

**Affiliations:** Wageningen Food Safety Research (WFSR), Part of Wageningen University & Research, 6700 AE Wageningen, The Netherlands

**Keywords:** Food safety, Control monitoring, Ambient ionization mass spectrometry, Screening, Coated blade spray

## Abstract

The use of certain antibiotics in food-producing animals is allowed in Europe following Regulation (EU) 2017/625. However, use could result in antibiotic residues in foodstuffs of animal origin. Maximum residue limits (MRLs) are in place to protect consumers. For monitoring purposes, animal matrices are tested to verify their compliance with these MRLs. Initially, matrices of (slaughtered) food animals are screened, often using a microbiological assay. Faster screening tests for antibiotics would be an advantage for control laboratories. Therefore, the present study describes, for the first time, the use of coated blade spray (CBS) followed by direct mass spectrometry (MS) analysis for the screening of tetracyclines, sulfonamides, quinolones, and macrolides residues from the renal area of intact bovine kidneys. An optimized workflow using two different desorption/ionization solutions per blade allowed screening of target compounds within 1 min per sample. The proof-of-principle of the CBS-MS method is validated according to (EU) 2021/808, presenting CCβ screening values of 0.1 × MRL for 43 analytes, 0.5 × MRL for 4 analytes, and 2.5 µg kg^−1^ for the prohibited substance dapsone, respectively. The developed method was successfully applied to seven official control samples of bovine kidneys. One of these samples was found to be positive using the CBS-MS method, which was confirmed as a true positive by LC-MSMS analysis. The developed method demonstrates that CBS devices can directly extract and analyze kidney samples for food safety testing.

**Supplementary Information:**

The online version contains supplementary material available at 10.1007/s00216-024-05605-1.

## Introduction

In cattle breeding, antibiotics can be used to treat bacterial diseases. Therefore, antibiotic treatment of farm animals could result in antibiotic residues in foodstuffs of animal origin [1]. Consequently, to reduce antibiotic residues in food and to protect consumers, maximum residue limits (MRLs) per foodstuff of animal origin and withdrawal periods for antibiotics are established in the European Union [2]. Food safety laboratories perform screening and, if required, confirmatory analyses to determine if residues exceeding the established MRLs are present [1]. Screening of antibiotics of different families, i.e., tetracycline, sulfonamide, quinolone, macrolide, β-lactam, and aminoglycoside antibiotic groups, is widely performed by microbiological (multi-)plate screening assays due to their cost-efficiency and coverage of the broad antibiotic spectrum in a single test [3–5]. However, in practice, these microbiological assays are time-consuming due to overnight incubation, and non-specific as the assay only provides identification at the group level [1, 6]. When comparing the microbiological methods to liquid chromatography-tandem mass spectrometry (LC–MS/MS)–based screenings methods, improved sensitivity and selectivity can be observed for the latter [7, 8]. Nevertheless, the sample preparation and clean-up procedures before analysis are more extensive, compromising a time- and cost-effective screening method.

To improve the laboratory throughput, ambient ionization mass spectrometry (AIMS) use has increased during the last decade in food and forensic control laboratories [9–12]. AIMS techniques allow rapid, real-time, high-throughput, and in situ analysis of solids, liquids, and gases, which are characterized by their simplified sample preparation and sample introduction protocols, reduced costs, and analysis time. The AIMS technique, known as coated blade spray (CBS), combines sampling and sample treatment using a solid-phase microextraction (SPME) coating. It performs direct mass spectrometry analysis by desorption and subsequent ionization [13]. As CBS offers minimal sample preparation and short analysis time by eliminating chromatography, it substantially increases speed compared to microbiological and LC–MS/MS-based screening techniques. Moreover, the sword-shaped blade device offers an integrated sampling of biological matrices [14–16]. This approach could enable smart sampling, i.e., providing a cost-effective and efficient method of sampling food-related matrices with a minimal footprint during transport and minimal sample preparation requirements. Especially, the applicability of CBS to sample and analyze a wide variety of liquid and solid biological matrices [16] sets it apart from other AIMS techniques like gel loading tip spray ionization mass spectrometry (GLT-SI-MS) [12]. To date, CBS-MS has shown its potential in food control analysis for authenticity of honey [17], pesticides in fruit matrices [15], sulfonamides in milk [14], and mycotoxin deoxynivalenol in flour [18].

This study assesses the potential of CBS-MS for fast extraction, screening, and identification of tetracycline, sulfonamide, quinolone, and macrolide antibiotics in kidney samples. The ultimate goal was to perform a direct kidney extraction by sticking the coated blade into the kidney, followed by direct measurement by MS. To reach this goal, a CBS-MS measurement method was developed for kidney fluid, whereby the most critical working parameters were evaluated and discussed. The developed CBS-MS method was validated according to (EU) 2021/808. The applicability of the CBS-MS technique for direct kidney extraction by the coated blade was demonstrated by identifying antibiotics in the renal area of an anonymized intact bovine kidney sample from the Dutch National Residue Control Plan.

## Experimental section

### Chemicals and standards

Ultra LC–MS grade methanol and isopropanol were obtained from Actu-All (Oss, The Netherlands). Formic acid (FA) was purchased from VWR International (Darmstadt, Germany). Ammonium acetate was purchased from Sigma-Aldrich (St. Louis, MO, USA). Purified water was prepared using a Milli-Q system at a resistance of at least 18.2 MΩ·cm (Millipore, Billerica, MA, USA). Analytical reference standards with certificates of analysis and purities of ≥ 90%, unless mentioned otherwise, of chlortetracycline, oxytetracycline, tetracycline, ciprofloxacin, danofloxacin, difloxacin, enrofloxacin, flumequine, marbofloxacin, nalidixic acid, norfloxacin, oxolinic acid, sarafloxacin (purity 89%), erythromycin, josamycin, lincomycin, spiramycin, tiamulin, tylosin (purity 85.7%), valnemulin, dapsone, sulfacetamide, sulfachlorpyridazine, sulfadimethoxine, sulfadimidine, sulfadoxine, sulfamerazine, sulfamethizole, sulfamethoxazole, sulfamethoxypyridazine, sulfamoxole, sulfaphenazole, sulfapyridine, sulfaquinoxaline, sulfathiazole, sulfisoxazole, and trimethoprim were purchased from Sigma-Aldrich (St. Louis, MO, USA). Neospiramycin I (87.4%), pirlimycin, and natamycin were purchased from Toronto Research Chemicals (Toronto, ON, Canada). Doxycycline and sulfadiazine were purchased from Council of Europe (EDQM, Strasbourg, France). Gamithromycin (purity 85.3%) and tulathromycin were purchased from Santa Cruz Biotechnology (Dallas, TX, USA). Tilmicosin (purity 85%) was purchased from Dr. Ehrenstorfer GMBH (Augsburg, Germany), tylvalosin (purity 65.9%) at ECO Animal Health (London, UK), tildipirosin from MSD Animal Health (Boxmeer, The Netherlands), and sulfamonomethoxine at TCI Europe (Zwijndrecht, Belgium). The internal standards of caffeine-^13^C was purchased from Sigma-Aldrich (St. Louis, MO, USA). A mixed solution of reference standards that enables spiking at the different MRLs was prepared at 60/200/10/10 mg L^−1^ (tetracyclines, quinolones, macrolides, sulfonamides) in MeOH.

### Samples and sample analysis

Bovine kidney samples were provided by the Dutch Food Safety Authority (NVWA) and originated from the routine monitoring program in The Netherlands. Bovine kidney samples used during validation were obtained from multiple bovine animals of different age and gender which were predetermined blank for antibiotic residues.

#### Kidney fluid

Kidney fluid was prepared by placing 10 g ± 0.5 g of homogenized kidney in a 50-mL tube and heating it in a water bath set to 80 °C for 10 min. Afterwards, the mixture was centrifuged at 2700 g for 10 min, and the supernatant was used for further experiments.

Prior to CBS sampling, all coated blades were preconditioned using methanol, followed by water. During validation studies, the internal standard caffeine-^13^C (25 µg L^−1^) was included in the preconditioning step with water. Sampling of kidney fluid was performed by placing the blade in an aliquot of 500 µL of blank or fortified kidney fluid sample and vortexing at 2700 rpm for 60 s. Matrix contaminants were removed by washing the blades with 500 µL of 5% methanol in water. Unless mentioned otherwise, desorption and ionization were performed using 10 µL of methanol:water (95:5 v/v + 12 mM of ammonium acetate) followed by re-analysis of the same blade using 10 µL methanol:water:formic acid (90:10:0.1 v/v/v) to cover the complete scope of 48 antibiotic substances.

#### Kidney sampling

The sampling procedure is similar to the method used for the New Dutch Kidney test (NDKT) with the slight modification of using coated blades for collection of tissue fluid by absorption [19, 20]. In short, an incision was made in the bovine kidney, coated blades were inserted and placed on the interface of the medulla, and renal pelvis fluid was collected by absorption. Blades were left in the renal medulla for 60 s, unless mentioned otherwise. Following sampling, blades were carefully cleaned with a Kimwipe tissue to remove any biological residue and washed in 500 µL of 5% methanol in water. Further analysis parameters were similar to the analysis of kidney fluid.

### Instrumentation

**Fig. 1:**
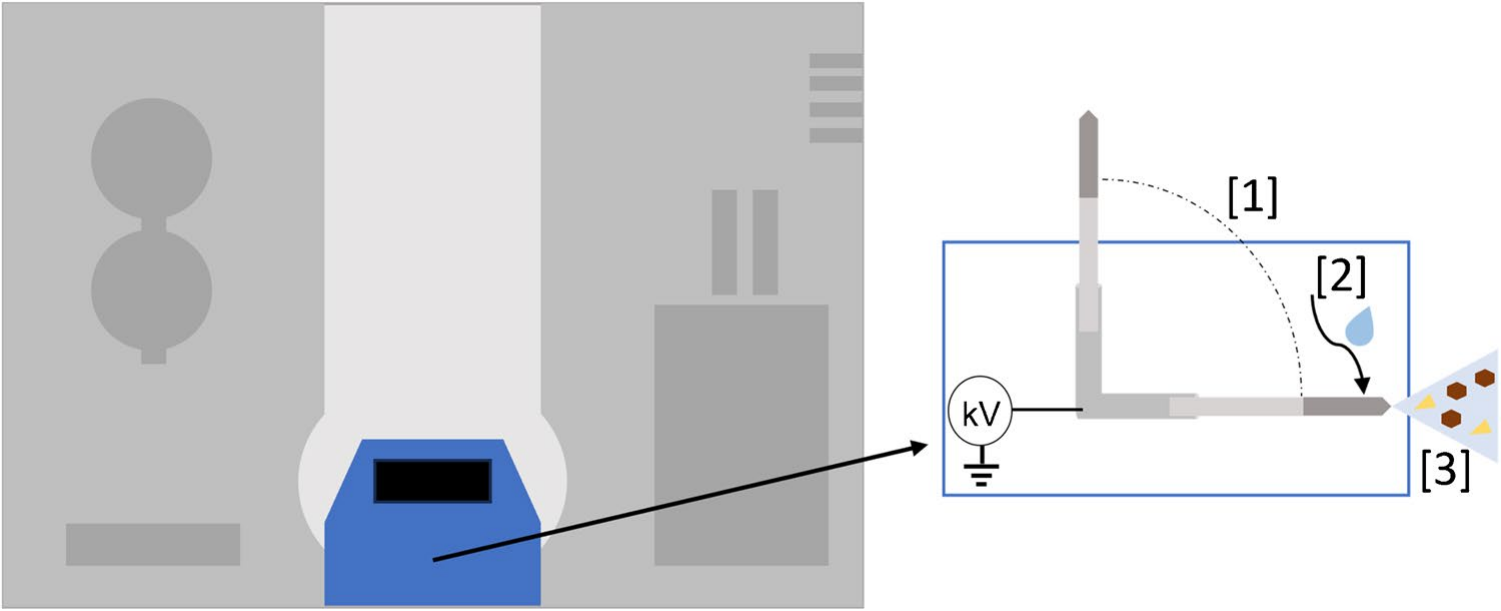
A schematic overview of the coated blade spray interface connected to the mass spectrometer [1]. A blade is automatically horizontally positioned in front of the mass spectrometer inlet, ensuring reproducible blade placement, [2] a set volume of spray solvent is automatically applied on the coated surface of the blade and left to desorb for a set amount of seconds (elution time), and [3] the high voltage supplied by the mass spectrometer is applied for a set amount of seconds (spray time) on the blade resulting in electrospray ionization. Finally, after spraying of the blade, the blade remains in the horizontal position for a set amount of seconds (drying time) and the mass spectrometer inlet is cleaned using a jet of organic solvents for a set amount of seconds (cleaning time)

Coated blades were coated with hydrophilic-lipophilic balance (HLB) stationary-phase material obtained from Restek (Bellefonte, PA, USA). All experiments were performed on a QB Sciex Quadrupole-trap 6500 mass spectrometer, equipped with an experimental research prototype blade spray ionization interface provided by Restek (Bellefonte, PA, USA). A schematic overview is presented in Fig. 1. The elution time was set to 10 s, the spray time to 20 s, and the drying and cleaning time to 18 and 10 s, respectively. A 20-s spray time was chosen to allow 60 scans of each compound transition at the selected dwell time of 3 ms. During the cleaning step, after blade analysis, the prototype interface automatically cleans the MS inlet by applying a solvent jet of methanol:isopropanol (50:50, v/v%) wash solution to avoid any cross-contamination risk between samples. Positive ionization and multiple-reaction monitoring (MRM) modes were used during all analyses (see supplementary information Table S1 for the monitored transitions and optimized settings).

CBS-MS/MS data was processed using the vendor-independent open-source software Skyline (version 22.2) [21]. To correctly integrate the whole area of the obtained chronograms, the peak boundaries for all samples and all transitions for each series were set to a start time of 0.1 and an end time of 0.8 min using the export and import peak boundaries option.

### CBS-MS/MS method validation

Since CBS-MS/MS does not determine a retention time, it is not possible to validate CBS as a confirmatory method. Therefore, it was validated as a qualitative screening method according to (EU) 2021/808 [22]. Maximum residue limits (MRLs) per foodstuff of animal origin and withdrawal periods for antibiotics are established in the European Union as shown in Table S2. Kidney fluid samples prepared from 21 different bovine kidneys were analyzed as blanks and fortified at 0.1 × MRL and 0.5 × MRL concentrations. The average signal for a compound in the blank samples (*n* = 21) was considered the noise level. The fortification level at which a compound could be detected with a signal-to-noise (S/N) > 3 for at least one transition in ≥ 95% of the fortified samples was considered the CCβ screening.

## Results and discussion

Currently, there are no in situ options to fortify intact kidney organs with our compounds of interest other than to destruct, homogenize, and fortify the organ tissue [1, 23, 24]. The only current option to obtain an incurred intact kidney organ is by performing animal studies. Out of practical and animal welfare considerations, the method development, optimization, and validation were performed in spiked kidney fluid, the isolated fluid from the tissue homogenate obtained by a brief heating step followed by centrifugation. As kidney fluid allowed comparison with MRL values by spiking with standards, it is assumed that antibiotic concentrations in kidney fluid would approximate those in intact kidney tissue [3, 20, 25, 26].

### Optimization of CBS-MS/MS conditions

**Fig. 2:**
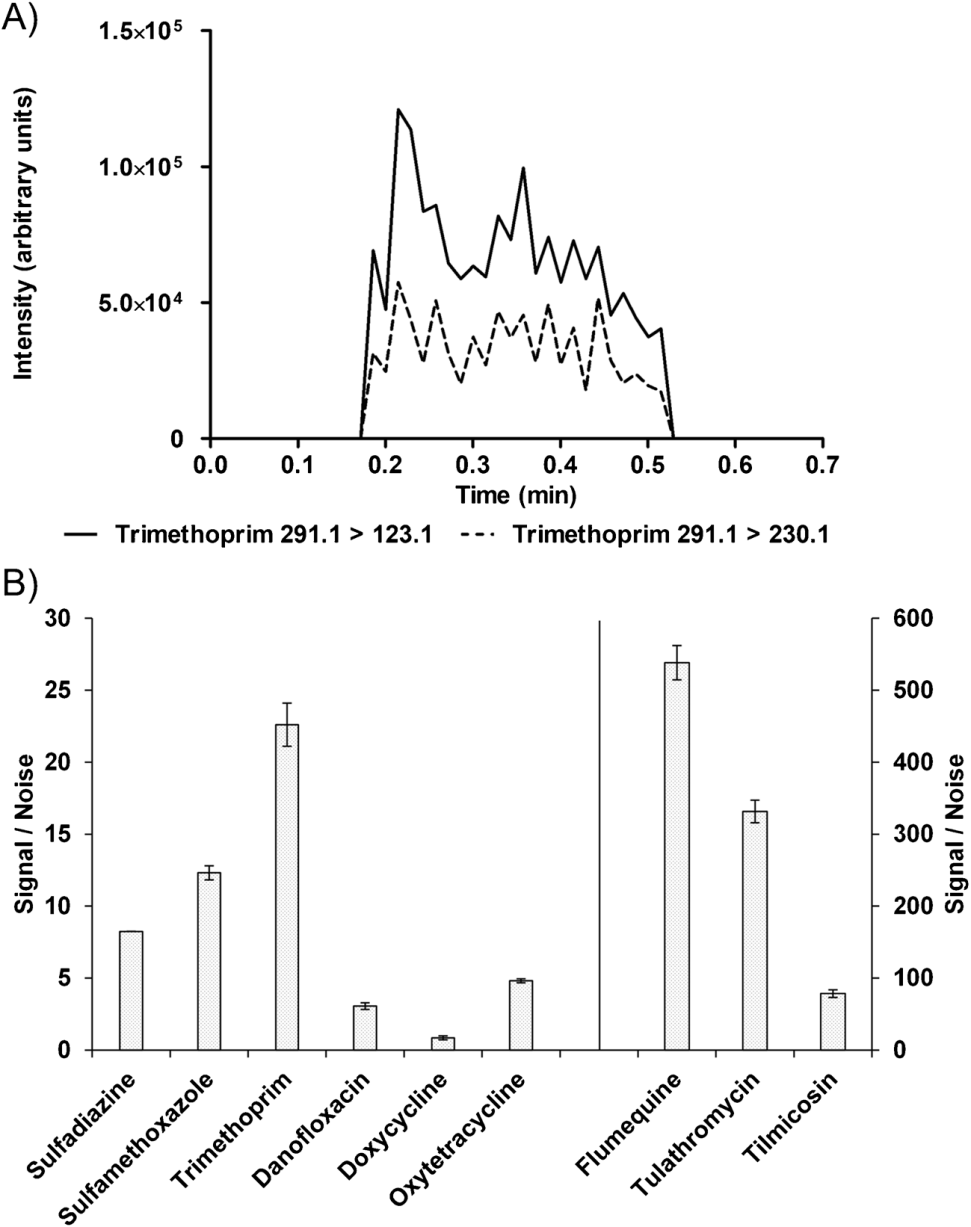
**A** Chronogram example of trimethoprim spiked in kidney fluid at 0.5 × MRL sprayed with methanol:water (95:5 v/v + 12 mM ammonium acetate) analyzed while monitoring a total of 107 transitions. **B** The signal-to-noise constructed from the main diagnostic ion of antibiotic substances sulfadiazine, sulfamethoxazole, trimethoprim, danofloxacin, doxycycline, oxytetracycline, flumequine, tulathromycin, and tilmicosin spiked in kidney fluid at 0.5 × MRL (*n* = 2) while monitoring a total of 107 transitions

The experimental research prototype blade spray ionization interface allowed for reproducible placement, desorption, and ionization of the coated blades (Fig. 1). A spray time of 20 s is used for each measurement. During these 20 s, a total of 107 MRM transitions, corresponding to 48 antibiotics (Table S1) from tetracyclines, sulfonamides, quinolones, and macrolides, are evaluated based on the signal intensity and S/N is measured. As shown in Fig. 2, when all 48 substances were analyzed simultaneously, they showed good performance in terms of signal/noise. These results agree with the findings from Kasperkiewicz et al. (2019) [27], which concluded that different dwell times at a constant spray time had no significant effect.

### Optimization of desorption solvent

**Fig. 3:**
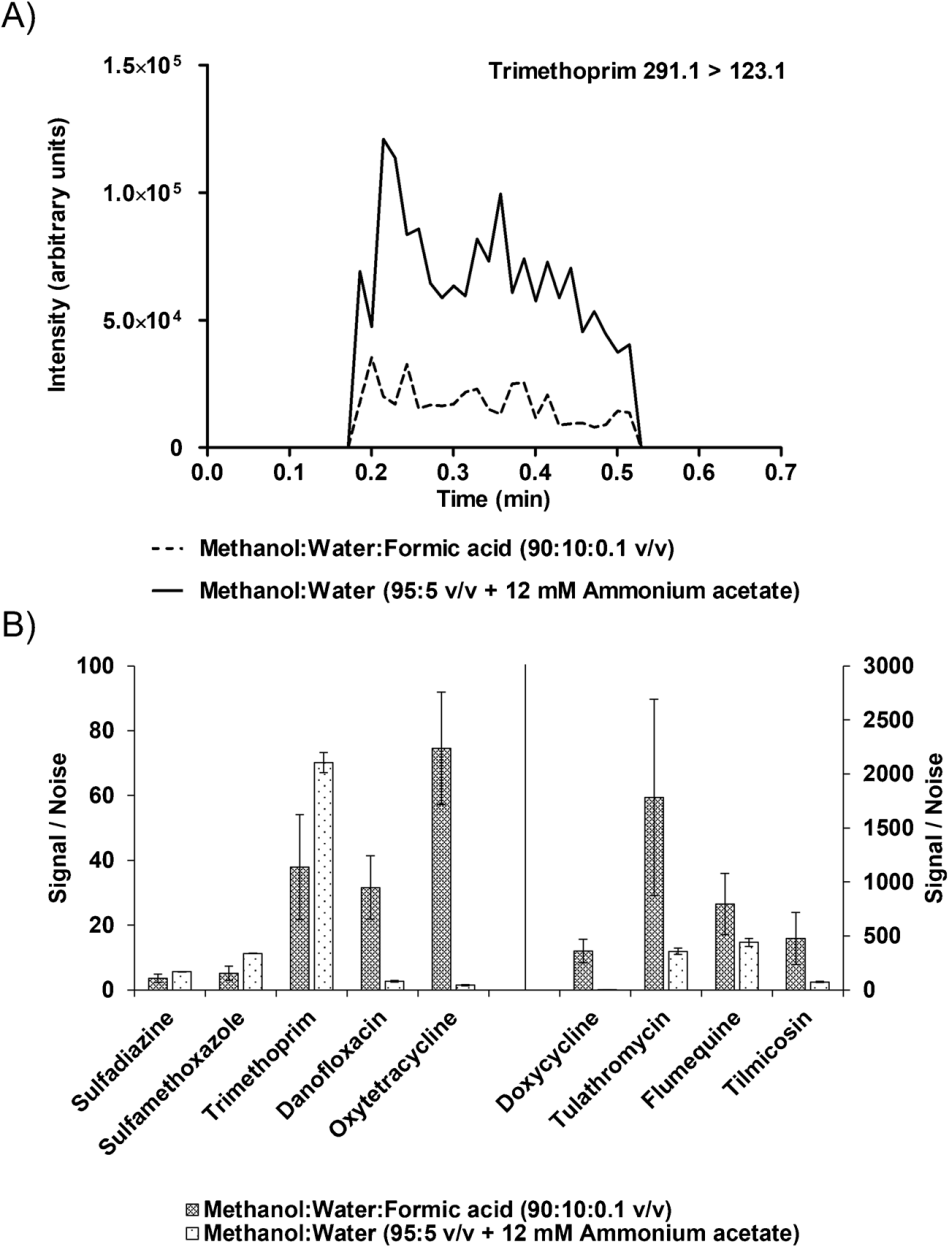
Comparison of spray solvents methanol:water:formic acid (90:10:0.1 v/v) and methanol:water (95:5 v/v + 12 mM ammonium acetate) on **A** the reconstructed chronograms of trimethoprim and **B** the signal-to-noise of antibiotic substances sulfadiazine, sulfamethoxazole, trimethoprim, danofloxacin, oxytetracycline, doxycycline, tulathromycin, flumequine, and tilmicosin spiked in kidney fluid at 0.5 × MRL (*n* = 2)

Desorption solvents in CBS-MS applications are required to desorb compounds from the coated surface and enable electrospray ionization [16, 28]. Therefore, the desorption solvent should be optimized per CBS-MS application. Different CBS-MS spray solvents using combinations of methanol, water, and a modifier were evaluated for all included antibiotics. Acetonitrile as an organic solvent was not evaluated, as earlier conducted research demonstrated poor compatibility with CBS-MS [14]. The area under the curve of the main diagnostic ion, the S/N, and the amount of variation (CV) were taken as evaluation criteria (Fig. 3). For visualization purposes, two antibiotics per individual class at 0.5 × MRL, being sulfadiazine, sulfamethoxazole, flumequine, danofloxacin, tilmicosin, doxycycline, oxytetracycline, and trimethoprim are displayed in figures below.

As can be observed in Fig. 3, the results showed that adding 0.1% formic acid improved the S/N compared to methanol:water:ammonium acetate, but resulted in a high variation between measurements (*n* = 2) for danofloxacin, tulathromycin, flumequine, and tilmicosin. Using the spray solvent of methanol:water:ammonium acetate significantly improved the repeatability, demonstrating CVs of 0.3%; 0.4%; 8%; 4%; 9%; 7%; 6%; and 12% for sulfadiazine, sulfamethoxazole, trimethoprim, danofloxacin, oxytetracycline, tulathromycin, flumequine, and tilmicosin, respectively.

While the spray solvent methanol:water:ammonium acetate significantly reduced the S/N of danofloxacin, tulathromycin, and tilmicosin at 0.5 × MRL, their respective S/N values remained above 3. The spray solvent consisting of methanol:water:ammonium acetate also had limited compatibility for the analysis of tetracyclines, as improved signal intensity and S/N were obtained using methanol:water:formic acid (90:10:0.1 v/v/v%) (Fig. 3). Consequently, methanol:water:ammonium acetate was selected as the optimal spray solvent for analyses for sulfonamides, quinolones, and macrolides, while methanol:water:formic acid (90:10:0.1 v/v/v%) was applied for tetracyclines as the spray solvent.

**Fig. 4:**
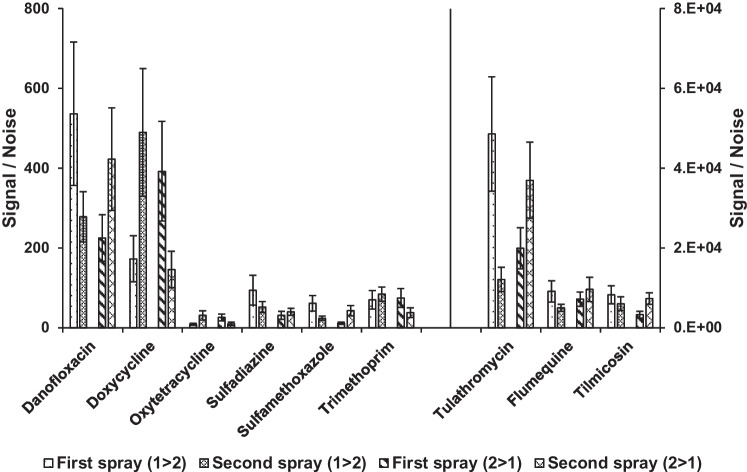
The effect of using two sequential spray solvents in alternating orders (1 > 2) and (2 > 1) per individual blade on the signal-to-noise of antibiotic substances danofloxacin, doxycycline, oxytetracycline, sulfadiazine, sulfamethoxazle, trimethoprim, tulathromycin, flumequine, and tilmicosin at 0.5 × MRL (*n* = 5) (solvent 1 = methanol:water (95:5 v/v + 12 mM ammonium acetate), solvent 2 = methanol:water:formic acid (90:10:0.1 v/v/v%))

Since, for routine applications, it might be required to screen a sample for the whole selection of antibiotics, it is most convenient if a single blade can be analyzed twice with different spray solvents so the maximum sensitivity is reached for all compounds without needing multiple blades. This was tested by extracting both blank and fortified kidney fluid (0.5 × MRL) and analyzing the blades twice, first using methanol:water (95:5 v/v + 12 mM ammonium acetate) (solvent 1) and then using methanol:water:formic acid (90:10:0.1 v/v/v%) (solvent 2). Additionally, studies were performed the other way around to ensure that blades can be analyzed multiple times without losing sensitivity. Figure 4 shows that the sensitivity for each desorption/spray experiment is similar regardless of the spraying solvent order and that enough amount of the residues remain on the blade after the first analysis to perform multiple analysis on the same blades.

### Determination of screening target concentrations of antibiotics in kidney-drip

**Fig. 5:**
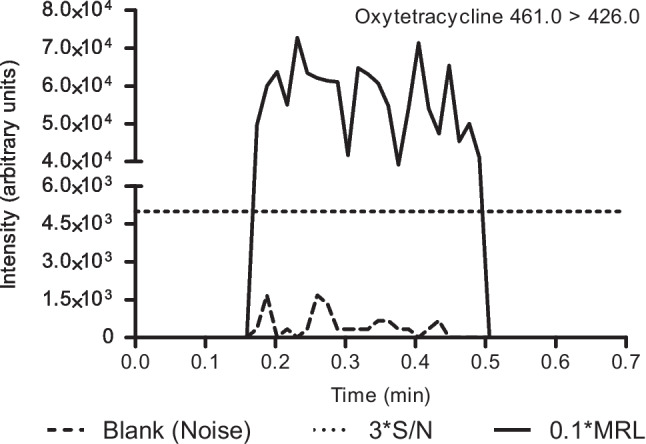
Chronograms from blank and spiked (0.1 * MRL) kidney fluid for oxytetracycline, including the 3 * S/N value based on the blank measurement

Under optimal CBS conditions, the screening target concentrations (STC) of antibiotics in kidney fluid were determined. Therefore, similar concentrations ranging from 0.1 × to 1 × MRL were spiked in kidney fluid. This fortified kidney fluid was consequently sampled using coated blade devices, which were washed with 5% methanol in water and directly analyzed by the developed CBS-MS/MS method. The lowest concentration demonstrating an S/N > 3 was chosen as the STC per individual antibiotic for further validation as a qualitative screening method (see Fig. 5). A total of 43 antibiotic substances could be detected at 0.1 × MRL. Sulfacetamide, sulfachloropyridazine, sulfaphenazole, and sulfaquinoxaline could be detected at 0.5 × MRL. Similar elevated detection levels for these sulfonamides have been demonstrated by Bogialli et al. in a LC–MS/MS method [29] and by Jager et al. in a CBS-MS application [14]. For dapsone, there is no MRL; the STC was determined to be at a concentration of 2.5 µg kg^−1^.

### Validation

The developed CBS-MS/MS method for tetracyclines, sulfonamides, quinolones, and macrolides was validated for qualitative screening of bovine kidneys according to (EU) 2021/808 [22]. In Table 1, the calculated CCβ screening values of the targeted antibiotic substances are shown. As can be observed in Table 1, all compounds can be measured at levels lower than the established MRL in kidney. In one case, the blade failed to spray; i.e., no caffeine-^13^C signal was observed. Therefore, this measurement was omitted from the validation study. Consequently, in practice, a kidney sample is not sampled singular, but at least by two blades.

**Table 1:** Validation results of antibiotic screening in kidney fluid using CBS-MS/MS

Compound	Optimal spray solvent*	CCβ screening (µg kg^−1^)	Equivalence in MRL bovine kidney	S/N > 3 at lowest validated level**
Danofloxacin	1	20	0.1 ×	20/20
Dapsone	1	2.5	− ***	20/21
Difloxacin	1	60	0.1 ×	20/20
Flumequine	1	100	0.1 ×	20/20
Gamithromycin	1	10	0.1 ×	19/20
Josamycin	1	20	-****	20/20
Lincomycin	1	150	0.1 ×	20/20
Marbofloxacin	1	15	0.1 ×	20/20
Nalidixic acid	1	10	-****	19/20
Natamycin	1	10	-****	19/20
Neospiramycin I	1	30	0.1 ×	20/20
Norfloxacin	1	10	-****	20/20
Oxolinic acid	1	15	0.1 ×	20/20
Pirlimycin	1	40	0.1 ×	20/20
Sarafloxacin	1	10	-****	19/20
Spiramycin	1	30	0.1 ×	20/20
Sulfaquinoxaline	1	50	0.5 ×	21/21
Sulfacetamide	1	50	0.5 ×	20/20
Sulfachloropyridazine	1	50	0.5 ×	20/20
Sulfadiazine	1	10	0.1 ×	19/20
Sulfadimethoxine	1	10	0.1 ×	20/20
Sulfadimidine	1	10	0.1 ×	20/20
Sulfadoxine	1	10	0.1 ×	20/20
Sulfamerazine	1	10	0.1 ×	19/20
Sulfamethizole	1	10	0.1 ×	19/20
Sulfamethoxazole	1	10	0.1 ×	19/20
Sulfamethoxypyridazine	1	10	0.1 ×	20/20
Sulfamonomethoxine	1	10	0.1 ×	20/20
Sulfamoxole	1	10	0.1 ×	20/20
Sulfaphenazole	1	50	0.5 ×	20/20
Sulfapyridine	1	10	0.1 ×	19/20
Sulfathiazole	1	10	0.1 ×	19/20
Sulfisoxazole	1	10	0.1 ×	20/20
Tiamulin	1	10	-****	20/20
Tildipirosin	1	300	0.1 ×	20/20
Tilmicosin	1	100	0.1 ×	20/20
Trimethoprim	1	5	0.1 ×	19/20
Tulathromycin	1	300	0.1 ×	20/20
Tylosin	1	10	0.1 ×	20/20
Tylvalosin	1	5	0.1 ×	20/20
Chlortetracycline	2	60	0.1 ×	21/21
Ciprofloxacin	2	20	0.1 ×	21/21
Doxycycline	2	60	0.1 ×	21/21
Enrofloxacin	2	20	0.1 ×	21/21
Erythromycin	2	20	0.1 ×	21/21
Oxytetracycline	2	60	0.1 ×	21/21
Tetracycline	2	60	0.1 ×	21/21
Valnemulin	2	10	0.1 ×	21/21

*1, MeOH:water (95:5 v/v + 12 mM ammonium acetate); 2, MeOH:water:FA (90:10:0.1 v/v/v)

**For the sample set using solvent 1 at 0.1 × MRL, 1 blade failed to spray and was omitted from the validation study

***Prohibited substance (table 2 Regulation (EU) 37/2010 [2]

****No MRL set in bovine kidney (Regulation (EU) 37/2010 [2]

### Internal standard addition when sampling intact kidney organ tissue

The ultimate objective was to insert the coated blades into a kidney, and conduct a direct extraction followed by CBS measurement to rapidly detect and identify antibiotic substances. During the direct coated blade extraction process in the kidney, including an internal standard is not feasible. However, CBS-MS applications frequently employ internal standards to correct recovery losses and signal fluctuations [14, 15, 28, 30, 31]. While isotopic labeled internal standards are preferably included during the matrix sampling, this is impossible when intact kidney organs are sampled. In an earlier experiment involving bovine urine (Figure S1), the effect of correcting acquired signal by using an internal standard was tested by adding an internal standard solution at four different stages of blade analysis: (1) preconditioning the internal standards on the blade before urine sampling, (2) including internal standards in a buffer solution during urine sampling, (3) incorporating internal standards into the washing solvent used after urine sampling, and (4) including internal standards in the spray solvent. The best results, in terms of corrections using internal standards during CBS measurements, resulting in better linearity and repeatability, were obtained when an internal standard solution with a buffer was included during urine sampling. However, promising results in terms of correction of fluctuations during CBS analysis were also obtained when blades were loaded with the internal standard before sampling. Simultaneously, the loading of blades before any sampling covers both the sampling and desorption/ionization steps. The signal correction strategies developed in bovine urine experiments demonstrate the possibility of preconditioning internal standard solutions on the blade before sampling of a biological matrix. Therefore, the loading of blades with the internal standard before sampling was used in the direct kidney sampling experiments. In this application, the internal standard was not primarily used for signal correction but for quality control purposes to determine if a blade measurement was successfully measured. As the internal standard solution would be preconditioned on the blade, prior to sampling of a kidney, the use of isotopically labelled antibiotic internal standards was omitted as this would result in contamination of the bovine kidney that could interfere with potential follow-up confirmatory testing. Therefore, the use of caffeine-^13^C was selected.

### Analysis of a panel of intact organ samples

**Fig. 6:**
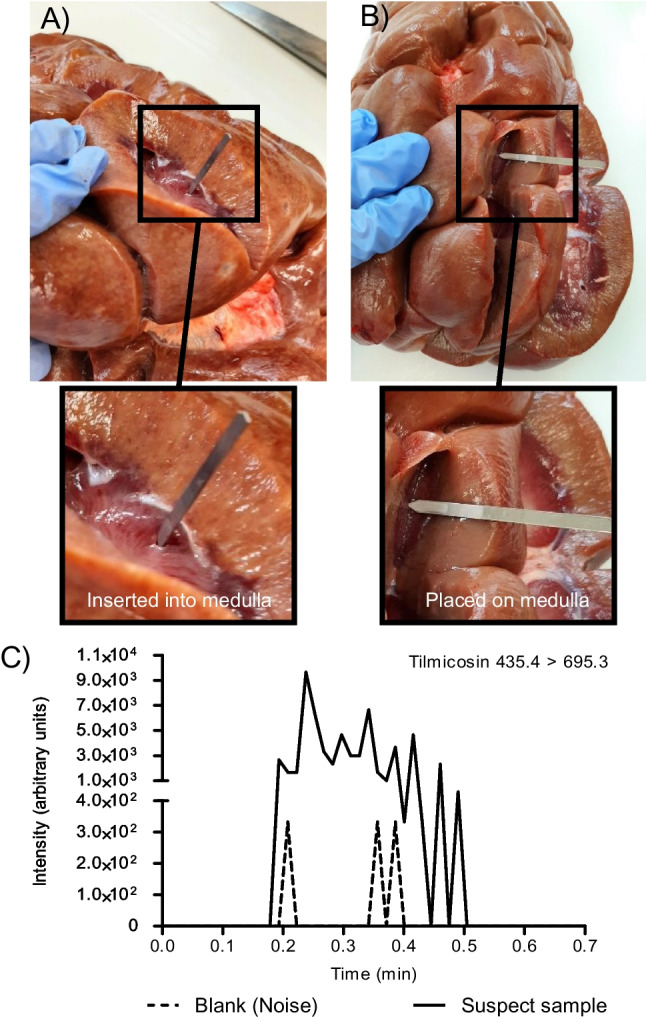
Sampling of intact bovine kidney organs using coated blades. **A** One blade is inserted into medulla’s interface. **B** A second blade is laid on the medulla’s interface. **C** Following CBS-MS/MS analysis, the chronograms of tilmicosin in the suspect sample and a blank (noise) are reconstructed, demonstrating the presence of tilmicosin

As a proof of concept, a total of seven intact bovine kidneys were sampled using two coated blades. One blade was inserted in the kidney (Fig. 6A), and the other was laid on the medulla’s interface (Fig. 6B). Out of seven sampled blades, one inserted and one laid blade originating from the same bovine kidney sample demonstrated an increase of 75 and 45 S/N ratio for tilmicosin, respectively. Additionally, the reconstructed chronograms from the positive screened kidney samples were compared against reconstructed chronograms from a negative control kidney sample, which was included during the analysis (Fig. 6C). As can be observed, the S/N deviation and the reconstructed chronograms demonstrated the presence of tilmicosin residues in the bovine kidney. Projecting the S/N ratio from this real-life example on the validation results for tilmicosin at 0.1 × MRL (100 µg kg^−1^) with minimum and maximum S/N ratios of 12 and 223 (average S/N ratio of 101) clearly indicates the presence of tilmicosin at or above the 0.1 × MRL level. The encountered differences in S/N ratios between the inserted and laid blade could originate from differences in renal pelvis fluid absorbance that correlate with the amount of moisture present, similar to e.g. NAT screening [20]. Also differences in contact between the renal tissue and the blade surface could differ and contribute to these differences.

Furthermore, the relative ion ratio between the practical sample and a kidney fluid sample spiked at 1 × MRL analyzed in the same series was calculated at 6%, well within the ± 40% relative deviation set in Regulation 2021/808 and could therefore be identified as tilmicosin [22]. Following these findings, the bovine kidney sample was submitted for confirmatory LC–MS/MS analysis, confirming the presence of tilmicosin at a concentration (338 µg kg^−1^) lower than the set MRL.

The other real-life samples were analyzed with direct CBS extraction and were determined to be blank, which was confirmed by either the performed post-screening test [26] or confirmatory LC–MS/MS analysis. Of course, for a reliable demonstration across the wider panel of included antibiotics, the panel of tested (positive) samples should be expanded. Nevertheless, these preliminary real-life examples demonstrate the potential of kidney sampling using coated blades, providing rapid substance identification.

## Conclusions

Efficient residue monitoring in food safety–related matrices such as kidneys is an important part of any monitoring program. Introducing ambient ionization techniques such as CBS has enabled efficient screening with the benefit of mass spectrometry’s selectivity and sensitivity. The present study used coated blades to screen for antibiotic residues in intact bovine kidneys. Additionally, experiments were performed to determine that the CBS-MS/MS method’s sensitivity remained within the established scope while monitoring more than 100 MRM transitions. Furthermore, it was demonstrated that blades could be analyzed multiple times without an apparent loss of sensitivity, meaning that the whole selection of compounds can be analyzed using their respective optimal spray solvent to maximize sensitivity while only needing a single blade. The method was successfully validated as a screening method according to (EU) 2021/808, and the CCβ screening was found to be at or below 0.5 * MRL for all studied compounds. Ultimately, using coated blades, the CBS method demonstrated its efficacy in extracting and identifying tilmicosin in an intact bovine kidney. This underscores the method’s capacity for efficient and fast antibiotic residue screening of intact kidneys.

## Supplementary Information

Below is the link to the electronic supplementary material.

Supplementary file1 (DOCX 135 KB)
