# Supplementary material for: Rapid identification of antibiotic residues in bovine kidney using coated blade spray-mass spectrometry
Source: Anal Bioanal Chem. 2024 Oct 22;416(29):7031–41. doi: 10.1007/s00216-024-05605-1 (PMC11579169; doi:10.1007/s00216-024-05605-1)
Supplement: Supplementary file 1 — Supplementary file1 (DOCX 135 KB) [file 216_2024_5605_MOESM1_ESM.docx]

**Supplementary material**

**Rapid identification of antibiotic residues in bovine kidney using Coated Blade Spray-Mass Spectrometry**

Josha Jager, Sjors Rasker, Ane Arrizabalaga-Larrañaga, Rita Boerrigter-Eenling, Michel Rapallini, Marco Blokland

Wageningen Food Safety Research, Wageningen University and Research, Akkermaalsbos 2, 6708 WB Wageningen, the Netherlands

* Corresponding author: Josha Jager

E-mail: [josha.jager@wur.nl](mailto:josha.jager@wur.nl)

Table of Contents

[**Supplementary Tables** 2](#_Toc179184666)

[**Table S1:** MRM transition list used for CBS-MS/MS analysis of antibiotics in kidney. 2](#_Toc179184667)

[**Table S2:** European Maximum Residue Limits of antibiotics in bovine kidney 5](#_Toc179184668)

[**Supplementary Figures** 7](#_Toc179184669)

[**Figure S1:** The effect on linearity and repeatability by internal standard correction at four different stages of blade analysis, (1) preconditioning the internal standards on the blade before urine sampling, (2) including internal standards in a buffer solution during urine sampling, (3) incorporating internal standards into the washing solvent used after urine sampling, and (4) including internal standards in the spray solvent. 7](#_Toc179184670)

# **Supplementary Tables**

## **Table S1:** MRM transition list used for CBS-MS/MS analysis of antibiotics in kidney.

| **Name** | **Q1** | **Q3** | **Dwell (ms)** | **Collision Energy (CE)** | **Collision Cell Exit Potential (CXP)** | **Declustering Potential (DP)** | **Entrance Potential (EP)** |
| --- | --- | --- | --- | --- | --- | --- | --- |
| Sulfacetamide | 215.1 | 155.9 | 3 | 23 | 12 | 41 | 10 |
| Sulfacetamide* | 215.1 | 92.0 | 3 | 27 | 10 | 41 | 10 |
| Nalidixic acid* | 233.1 | 159.0 | 3 | 43 | 14 | 41 | 10 |
| Nalidixic acid | 233.1 | 187.0 | 3 | 33 | 16 | 41 | 10 |
| Sulfathiazole | 256.0 | 91.9 | 3 | 35 | 12 | 41 | 10 |
| Sulfathiazole* | 256.0 | 155.9 | 3 | 21 | 14 | 41 | 10 |
| Dapsone* | 249.0 | 155.9 | 3 | 19 | 14 | 51 | 10 |
| Dapsone | 249.0 | 107.9 | 3 | 29 | 12 | 51 | 10 |
| Sulfapyridine* | 250.0 | 155.9 | 3 | 23 | 14 | 51 | 10 |
| Sulfapyridine | 250.0 | 92.0 | 3 | 35 | 10 | 51 | 10 |
| Sulfadiazine* | 251.0 | 156.0 | 3 | 21 | 14 | 46 | 10 |
| Sulfadiazine | 251.0 | 91.9 | 3 | 35 | 12 | 46 | 10 |
| Sulfamethoxazole* | 254.0 | 155.9 | 3 | 21 | 14 | 46 | 10 |
| Sulfamethoxazole | 254.0 | 92.0 | 3 | 27 | 12 | 46 | 10 |
| Oxolinic acid* | 262.0 | 215.9 | 3 | 39 | 16 | 36 | 10 |
| Oxolinic acid | 262.0 | 160.0 | 3 | 49 | 12 | 36 | 10 |
| Flumequine* | 262.0 | 201.9 | 3 | 43 | 16 | 36 | 10 |
| Flumequine | 262.0 | 126.0 | 3 | 61 | 8 | 36 | 10 |
| Sulfamerazine | 265.0 | 155.9 | 3 | 23 | 14 | 46 | 10 |
| Sulfamerazine* | 265.0 | 171.9 | 3 | 23 | 16 | 46 | 10 |
| Sulfamoxole* | 268.0 | 155.9 | 3 | 23 | 22 | 46 | 10 |
| Sulfamoxole | 268.0 | 92.0 | 3 | 35 | 12 | 46 | 10 |
| Sulfisoxazole* | 268.0 | 156.0 | 3 | 19 | 12 | 41 | 10 |
| Sulfisoxazole | 268.0 | 113.0 | 3 | 21 | 12 | 41 | 10 |
| Sulfamethizole* | 271.0 | 155.9 | 3 | 19 | 14 | 41 | 10 |
| Sulfamethizole | 271.0 | 92.0 | 3 | 37 | 12 | 41 | 10 |
| Sulfadimidine* | 279.0 | 186.0 | 3 | 23 | 16 | 66 | 10 |
| Sulfadimidine | 279.0 | 124.0 | 3 | 31 | 8 | 66 | 10 |
| Sulfamethoxypyridazine* | 281.0 | 156.0 | 3 | 23 | 12 | 66 | 10 |
| Sulfamethoxypyridazine | 281.0 | 108.0 | 3 | 39 | 14 | 66 | 10 |
| Sulfamonomethoxine* | 281.0 | 155.9 | 3 | 25 | 12 | 51 | 10 |
| Sulfamonomethoxine | 281.0 | 92.0 | 3 | 41 | 14 | 51 | 10 |
| Sulfachloropyridazine* | 285.0 | 155.9 | 3 | 21 | 10 | 36 | 10 |
| Sulfachloropyridazine | 285.0 | 91.9 | 3 | 39 | 10 | 36 | 10 |
| Trimethoprim | 291.1 | 123.1 | 3 | 25 | 25 | 36 | 10 |
| Trimethoprim* | 291.1 | 230.1 | 3 | 25 | 25 | 36 | 10 |

**Table S1 (cont.):** MRM transition list used for CBS-MS/MS analysis of antibiotics in kidney.

| **Name** | **Q1** | **Q3** | **Dwell (ms)** | **CE** | **CXP** | | **DP** | **EP** |
| --- | --- | --- | --- | --- | --- | --- | --- | --- |
| Sulfaquinoxaline | 301.0 | 155.9 | 3 | 23 | | 12 | 56 | 10 |
| Sulfaquinoxaline* | 301.0 | 92.0 | 3 | 41 | | 10 | 56 | 10 |
| Sulfadimethoxine* | 311.1 | 156.1 | 3 | 27 | 12 | | 41 | 10 |
| Sulfadimethoxine | 311.1 | 92.0 | 3 | 45 | 12 | | 41 | 10 |
| Sulfadoxine* | 311.1 | 155.9 | 3 | 25 | 12 | | 56 | 10 |
| Sulfadoxine | 311.1 | 107.9 | 3 | 33 | 14 | | 56 | 10 |
| Sulfaphenazole | 315.0 | 155.9 | 3 | 39 | 14 | | 51 | 10 |
| Sulfaphenazole* | 315.0 | 92.0 | 3 | 29 | 12 | | 51 | 10 |
| Sulfaphenazole | 315.0 | 108.0 | 3 | 37 | 8 | | 51 | 10 |
| Norfloxacin | 320.0 | 231.0 | 3 | 29 | 22 | | 61 | 10 |
| Norfloxacin* | 320.0 | 282.0 | 3 | 41 | 20 | | 61 | 10 |
| Ciprofloxacin* | 332.0 | 288.0 | 3 | 25 | 22 | | 66 | 10 |
| Ciprofloxacin | 332.0 | 231.0 | 3 | 49 | 20 | | 66 | 10 |
| Danofloxacin* | 358.0 | 255.0 | 3 | 55 | 20 | | 66 | 10 |
| Danofloxacin | 358.0 | 82.0 | 3 | 83 | 12 | | 66 | 10 |
| Enrofloxacin* | 360.1 | 286.0 | 3 | 47 | 22 | | 61 | 10 |
| Enrofloxacin | 360.1 | 316.0 | 3 | 27 | 24 | | 61 | 10 |
| Marbofloxacin* | 363.0 | 319.9 | 3 | 21 | 24 | | 56 | 10 |
| Marbofloxacin | 363.0 | 72.0 | 3 | 49 | 10 | | 56 | 10 |
| Tildipyrosin* | 368.0 | 637.5 | 3 | 18 | 26 | | 65 | 10 |
| Tildipyrosin | 368.0 | 98.0 | 3 | 23 | 22 | | 65 | 10 |
| Tildipyrosin | 368.0 | 174.2 | 3 | 18 | 12 | | 65 | 10 |
| Sarafloxacin* | 386.1 | 299.0 | 3 | 37 | 26 | | 81 | 10 |
| Sarafloxacin | 386.1 | 342.0 | 3 | 27 | 26 | | 81 | 10 |
| Sarafloxacin | 386.1 | 368.0 | 3 | 27 | 26 | | 81 | 10 |
| Difloxacin* | 400.1 | 299.0 | 3 | 39 | 24 | | 46 | 10 |
| Difloxacin | 400.1 | 356.0 | 3 | 27 | 26 | | 46 | 10 |
| Tulathromycin | 404.0 | 72.1 | 3 | 50 | 12 | | 40 | 10 |
| Tulathromycin* | 404.0 | 577.1 | 3 | 20 | 12 | | 40 | 10 |
| Lincomycin* | 407.1 | 126.1 | 3 | 33 | 12 | | 81 | 10 |
| Lincomycin | 407.1 | 359.0 | 3 | 25 | 32 | | 81 | 10 |
| Pirlimycin | 411.0 | 112.0 | 3 | 31 | 14 | | 76 | 10 |
| Pirlimycin* | 411.0 | 363.1 | 3 | 23 | 28 | | 76 | 10 |
| Spiramycin* | 422.2 | 174.1 | 3 | 27 | 14 | | 46 | 10 |
| Spiramycin | 422.2 | 101.3 | 3 | 27 | 14 | | 46 | 10 |
| Tilmicosin* | 435.4 | 695.3 | 3 | 25 | 12 | | 80 | 10 |
| Tilmicosin | 435.4 | 174.1 | 3 | 35 | 12 | | 80 | 10 |
| Tetracycline | 445.1 | 154.0 | 3 | 37 | 12 | | 56 | 10 |
| Tetracycline* | 445.1 | 410.0 | 3 | 27 | 12 | | 56 | 10 |
|  |  |  |  |  |  | |  |  |

**Table S1 (cont.):** MRM transition list used for CBS-MS/MS analysis of antibiotics in kidney.

| **Name** | **Q1** | **Q3** | **Dwell (ms)** | **CE** | **CXP** | **DP** | **EP** |
| --- | --- | --- | --- | --- | --- | --- | --- |
| Doxycycline | 445.2 | 98.0 | 3 | 60 | 12 | 40 | 10 |
| Doxycycline | 445.2 | 153.9 | 3 | 40 | 12 | 40 | 10 |
| Doxycycline* | 445.2 | 410.0 | 3 | 35 | 12 | 40 | 10 |
| Doxycycline | 445.2 | 428.0 | 3 | 25 | 12 | 40 | 10 |
| Oxytetracycline* | 461.0 | 426.0 | 3 | 27 | 12 | 61 | 10 |
| Oxytetracycline | 461.0 | 443.9 | 3 | 23 | 34 | 61 | 10 |
| Oxytetracycline | 461.0 | 154.0 | 3 | 33 | 13 | 60 | 10 |
| Oxytetracycline | 461.0 | 283.0 | 3 | 54 | 12 | 61 | 10 |
| Demeclocycline | 465.0 | 430.0 | 3 | 30 | 32 | 50 | 10 |
| Demeclocycline* | 465.0 | 448.0 | 3 | 25 | 32 | 50 | 10 |
| Chlortetracycline | 479.1 | 98.0 | 3 | 60 | 12 | 40 | 10 |
| Chlortetracycline | 479.1 | 154.0 | 3 | 38 | 12 | 40 | 10 |
| Chlortetracycline | 479.1 | 444.1 | 3 | 30 | 12 | 40 | 10 |
| Chlortetracycline* | 479.1 | 462.1 | 3 | 25 | 12 | 40 | 10 |
| Tiamulin* | 494.1 | 192.1 | 3 | 27 | 14 | 81 | 10 |
| Tiamulin | 494.1 | 119.0 | 3 | 55 | 8 | 81 | 10 |
| Valnemulin | 565.1 | 263.0 | 3 | 25 | 34 | 56 | 10 |
| Valnemulin* | 565.1 | 164.1 | 3 | 43 | 14 | 56 | 10 |
| Natamycin* | 666.3 | 503.2 | 3 | 15 | 13 | 40 | 10 |
| Natamycin | 666.3 | 648.3 | 3 | 17 | 13 | 40 | 10 |
| Neospiramycin I* | 699.4 | 174.1 | 3 | 27 | 14 | 60 | 10 |
| Neospiramycin I | 699.4 | 142.1 | 3 | 30 | 15 | 60 | 10 |
| Erythromycin* | 734.2 | 98.0 | 3 | 111 | 12 | 60 | 10 |
| Erythromycin | 734.2 | 158.0 | 3 | 45 | 14 | 60 | 10 |
| Gamithromycin* | 778.0 | 619.2 | 3 | 45 | 48 | 56 | 10 |
| Gamithromycin | 778.0 | 158.0 | 3 | 47 | 14 | 56 | 10 |
| Josamycin* | 828.1 | 174.0 | 3 | 41 | 14 | 56 | 10 |
| Josamycin | 828.1 | 109.0 | 3 | 83 | 16 | 56 | 10 |
| Tylosin* | 916.4 | 174.0 | 3 | 47 | 14 | 60 | 10 |
| Tylosin | 916.4 | 772.2 | 3 | 43 | 52 | 60 | 10 |
| Tylvalosin* | 1042.2 | 814.1 | 3 | 45 | 20 | 60 | 10 |
| Tylvalosin | 1042.2 | 174.0 | 3 | 30 | 13 | 60 | 10 |
| Caffeine-^13^C (IS) | 198.0 | 140.0 | 3 | 29 | 15 | 45 | 15 |

*Transition used for screening

## **Table S2:** European Maximum Residue Limits of antibiotics in bovine kidney

| **Compound** | **Compound group** | **MRL bovine kidney (µg kg^−1^)** |
| --- | --- | --- |
| Danofloxacin | Quinolone | 200 |
| Dapsone | Sulfonamide | -^*1^ |
| Difloxacin | Quinolone | 600^*2^ |
| Flumequine | Quinolone | 1000^*2^ |
| Gamithromycin | Macrolide | 100 |
| Josamycin | Macrolide | -^*5^ |
| Lincomycin | Macrolide | 1500 |
| Marbofloxacin | Quinolone | 150 |
| Nalidixic acid | Quinolone | -^*5^ |
| Natamycin |  | -^*3^ |
| Neospiramycin I | Macrolide | 300^*4^ |
| Norfloxacin | Quinolone | -^*5^ |
| Oxolinic acid | Quinolone | 150 |
| Pirlimycin | Macrolide | 400 |
| Sarafloxacin | Quinolone | -^*5^ |
| Spiramycin | Macrolide | 300^*4^ |
| Sulfaquinoxaline | Sulfonamide | 100 |
| Sulfacetamide | Sulfonamide | 100 |
| Sulfachloropyridazine | Sulfonamide | 100 |
| Sulfadiazine | Sulfonamide | 100 |
| Sulfadimethoxine | Sulfonamide | 100 |
| Sulfadimidine | Sulfonamide | 100 |
| Sulfadoxine | Sulfonamide | 100 |
| Sulfamerazine | Sulfonamide | 100 |
| Sulfamethizole | Sulfonamide | 100 |
| Sulfamethoxazole | Sulfonamide | 100 |
| Sulfamethoxypyridazine | Sulfonamide | 100 |
| Sulfamonomethoxine | Sulfonamide | 100 |
| Sulfamoxole | Sulfonamide | 100 |
| Sulfaphenazole | Sulfonamide | 100 |
| Sulfapyridine | Sulfonamide | 100 |
| Sulfathiazole | Sulfonamide | 100 |
| Sulfisoxazole | Sulfonamide | 100 |
| Tiamulin | Macrolide | -^*5^ |
| Tildipyrosin | Macrolide | 3000 |
| Tilmicosin | Macrolide | 1000 |
| Trimethoprim | Sulfonamide | 50 |
| Tulathromycin | Macrolide | 3000 |

**Table S2 (cont.):** European Maximum Residue Limits of antibiotics in bovine kidney

| **Compound** | **Compound group** | **MRL bovine kidney (µg kg^−1^)** |
| --- | --- | --- |
| Tylosin | Macrolide | 100 |
| Tylvalosin | Macrolide | 50 |
| Chlortetracycline | Tetracycline | 600 |
| Ciprofloxacin | Quinolone | 200^*6^ |
| Doxycycline | Tetracycline | 600 |
| Enrofloxacin | Quinolone | 200^*6^ |
| Erythromycin | Macrolide | 200 |
| Oxytetracycline | Tetracycline | 600 |
| Tetracycline | Tetracycline | 600 |
| Valnemulin | Macrolide | 100 |

^*1^ Prohibited substance in food producing animals

^*2^ MRL for all food producing animals is used

^*3^ No MRL required

^*4^ MRL is set on the summation of spiramycin and neospiramycin

^*5^ No MRL set in bovine kidney (Regulation (EU) 37/2010)

^*6^ MRL is set on the summation of ciprofloxacin and enrofloxacin

# **Supplementary Figures**


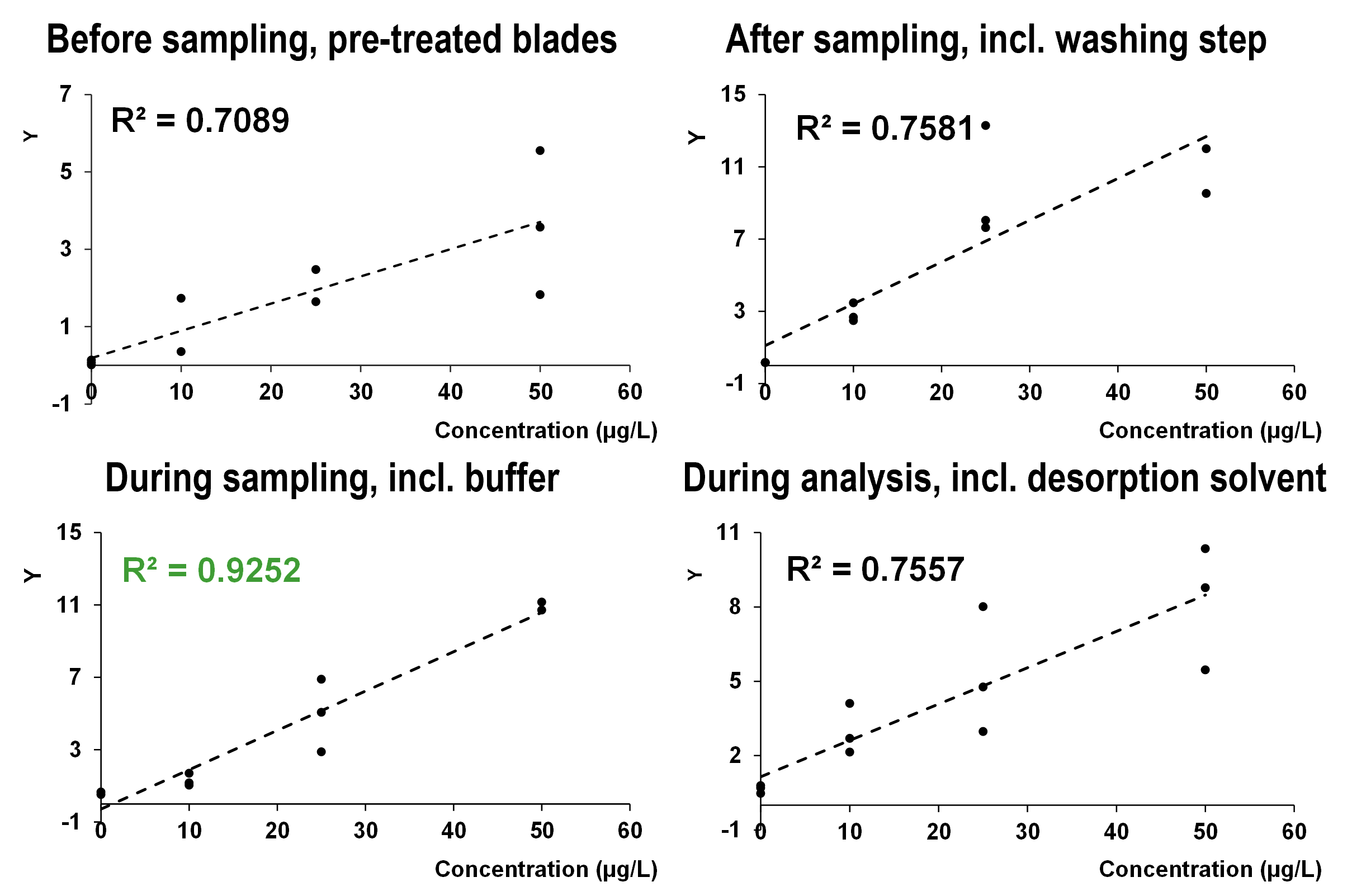


## **Figure S1:** The effect on linearity and repeatability by internal standard correction at four different stages of blade analysis, (1) preconditioning the internal standards on the blade before urine sampling, (2) including internal standards in a buffer solution during urine sampling, (3) incorporating internal standards into the washing solvent used after urine sampling, and (4) including internal standards in the spray solvent.
